# DIEER: Delay-Intolerant Energy-Efficient Routing with Sink Mobility in Underwater Wireless Sensor Networks

**DOI:** 10.3390/s20123467

**Published:** 2020-06-19

**Authors:** Kamran Latif, Nadeem Javaid, Imdad Ullah, Zeeshan Kaleem, Zafar Abbas Malik, Long D. Nguyen

**Affiliations:** 1 National Institute of Electronics, Islamabad 44000, Pakistan; zafarabbasmalik@gmail.com; 2 Department of Computer Science, COMSATS University Islamabad, Islamabad Campus, Islamabad 44000, Pakistan; nadeemjavaid@comsats.edu.pk; 3 Department of Information System, College of Computer Engineering and Sciences, Prince Sattam bin Abdulaziz University, Al-Kharj 11942, Saudia Arabia; i.ullah@psau.edu.sa; 4 The University of New South Wales (UNSW), School of Computer Science and Engineering (CSE), Sydney 2052, Australia; 5 Electrical and Computer Engineering Department, COMSATS University Islamabad, Wah Campus, Wah Cantt 47040, Pakistan; zeeshankaleem@gmail.com; 6 Institute of Research and Development, Duy Tan University, Da Nang 550000, Vietnam; nguyendinhlong1@duytan.edu.vn

**Keywords:** delay sensitive, under water WSN routing, energy-efficient routing, wireless sensor networks, sink mobility

## Abstract

Underwater Wireless Sensor Networks (UWSNs) are an enabling technology for many applications in commercial, military, and scientific domains. In some emergency response applications of UWSN, data dissemination is more important, therefore these applications are handled differently as compared to energy-focused approaches, which is only possible when propagation delay is minimized and packet delivery at surface sinks is assured. Packet delivery underwater is a serious concern because of harsh underwater environments and the dense deployment of nodes, which causes collisions and packet loss. Resultantly, re-transmission causes energy loss and increases end-to-end delay ($D_{E2E}$). In this work, we devise a framework for the joint optimization of *sink mobility*, *hold and forward mechanisms*, *adoptive depth threshold* ($d_{th}$) and *data aggregation with pattern matching* for reducing nodal propagation delay, maximizing throughput, improving network lifetime, and minimizing energy consumption. To evaluate our technique, we simulate the three-dimensional (3-D) underwater network environment with mobile sink and dense deployments of sensor nodes with varying communication radii. We carry out scalability analysis of the proposed framework in terms of network lifetime, throughput, and packet drop. We also compare our framework to existing techniques, i.e., Mobicast and iAMCTD protocols. We note that adapting varying $d_{th}$ based on node density in a range of network deployment scenarios results in a reduced number of re-transmissions, good energy conservation, and enhanced throughput. Furthermore, results from extensive simulations show that our proposed framework achieves better performance over existing approaches for real-time delay-intolerant applications.

## 1. Introduction

In recent years, the trend towards underwater environment exploration has gained significant attention due to the immense number of underwater applications such as environmental monitoring, ocean sampling, undersea exploration, assisted navigation, disaster prevention [1,2], mine reconnaissance, and pollution monitoring, and localization [3,4]. Typically, wireless sensors (nodes) are deployed in a targeted area to sense information of interest. The sensed information is then sent to surface sink(s) where it is properly interpreted [5]. Based on the interpreted data, experts or expert systems take necessary measures subject to task completion. The underwater nodes have more costly hardware than the terrestrial ones, and need relatively high transmit power to account for the harsh underwater channel conditions [6]. These nodes are prone to failure as they have limited battery power. Typically, the propagation delay in Underwater Wireless Sensor Networks (UWSNs) is five times larger than terrestrial sensor networks [7], the available bandwidth is highly limited, and Global Positioning System (GPS) is not available [8].

In addition to energy-efficient network operations, most of the underwater applications demand higher throughput and minimum end-to-end delay $\left(D_{E2E}\right)$. At the network layer, routing protocols play an important role in conserving node energy and improving throughput of the network. However, the uneven and drastic conditions of the ocean make the routing task highly challenging. Moreover, the lack of GPS in the underwater environment further complicates the process. In terms of location, the only available information underwater is depth of nodes. The authors in [9] use this information to route data from source(s) at higher depth to destination/sink at lower depth. However, the intermediate nodes are heavily involved in data-forwarding. To reduce forwarding load on relaying nodes, mobile sink is introduced in [10]. In the literature, mobile sink is also known as the courier node [11] and Autonomous Underwater Vehicle (AUV) [12]. Mobile sinks are equipped with high energy source and long-range communication capabilities. Mobile sinks move within the targeted network field and collect data from nodes.

In this research work, our main contributions are: the introduction of delay-intolerant routing protocol algorithm for applications such as navy battle surveillance, tsunami warnings etc.; the reduction of $D_{E2E}$ and maximization of throughput; mobile sink is used to further reduce nodal propagation delay and energy consumption; the adoption of varying ($d_{th}$) and data aggregation with pattern-matching technique further helps to reduce number of re-transmissions that ultimately conserve energy and enhances throughput. We use mobile sink in a three-dimensional (3-D) underwater network field to decrease the transmission distance between source and destination node. Thus, the usage of mobile sink reduces the propagation delay and enhances the throughput. Based on the availability of nodes in the vicinity of the source node, we optimize ($d_{th}$) between the source and forwarding node to reduce the number of transmissions and receptions. A reduced number of transmissions and receptions has two major benefits; minimized energy consumption and minimized nodal delays. Therefore, simulation results show that the overall proposed approach yields maximum throughput, minimal $D_{E2E}$, and maximized network lifetime.

The rest of the sections are organized as follows: Section 2 discusses the work done in this domain, Section 3 deals with energy-consumption model, Section 4 focuses on the calculation of optimal $d_{th}$, and Section 5 discusses the proposed DIEER protocol, Section 6 formulates network lifetime maximization and $D_{E2E}$ minimization models as linear programs, Section 7 discusses experimental results, and finally conclusions are presented in Section 8.

## 2. Related Work

It is a fact that every routing scheme cannot handle all the challenges and issues altogether [13]. Due to the unique characteristics of underwater conditions, different routing strategies are designed to address different challenges of UWSNs. Therefore, we categorize literature based on these techniques.

### 2.1. Energy-Efficient Focused Techniques

Depth-Based Routing (DBR) is the most popular technique in UWSNs, which is introduced in [14]. In this technique, nodes are deployed on the seabed that sense data, and nodes floating in the ocean layers work as relay nodes. Data travels from the seabed to sinks floating on the sea surface through relay nodes. Through this technique, a novel idea is proposed for location-free routing. However, imbalanced energy consumption of nodes is a problem; therefore, multiple variants of the technique are introduced for improvements. In Energy-Efficient Depth-Based Routing (EEDBR) Protocol for UWSNs [15], the authors overcome the imbalanced energy consumption of [14]. They modified the basic selection criteria of forwarder node presented in [14] by introducing Residual Energy (RE) factor for next hop selection. However, in this technique, RE is continuously changing and needs to be frequently updated among nodes, which itself is an energy-consumption activity. Therefore, balanced energy consumption issue persists in this technique. In [16], Z. Wadud et al. have proposed an energy-efficient and reliable protocol named as Energy Balanced Efficient and Reliable Routing (EBER). The authors considered the residual energy and the number of Potential Forwarding Nodes as a tool for energy conservation.

### 2.2. Void Node Avoidance Techniques

In [17], the authors proposed a scheme named Adaptive Transmission Range in WDFAD-DBR (ATR-WDFAD-DBR) and Cluster Based WDFAD-DBR (CBWDFAD-DBR). In this scheme, clusters are used to reduce the end-to-end delay and energy consumption. For void node avoidance, transmission ranges are adjusted to continue the communication process without any disruption. In [18], Khan et al. proposed a protocol that adapts to three types of networks depending on the node density. The parameters used in this work are packet delivery ratio and end-to-end delay, where the former is maximized and the latter is minimized. In [19], underwater sensors are given cubic spaces and geographically distributed in such framework. Forwarding nodes are selected using these geographic locations and packet delivery probability. Additionally, void nodes are also able to send their data to the mobile sink nodes for avoidance. Another work in [20] is introduced to avoid void nodes. In this work, every second hop of the packet is checked whether the state of the node at second hop is void or not. In case of void node, routing path is changed. In [21], network lifetime is in focus with the energy hole problem being overcome. In this work the network nodes are divided into wedge-like sectors and these sectors represent certain strengths of the sensor field by partitioning distance from sensor to sink in the form of coronas and deploying a transferring node in each of the corona. Jan et al. propose an algorithm for optimal energy consumption and hole alleviation in [22]. This algorithm focuses on increasing network lifetime, judicious distribution of Energy, and improvement in throughput.

### 2.3. Mobile Sink Assisted Techniques

In [10], the authors proposed an AUV-aided Underwater Routing Protocol (AURP). The proposed protocol takes the advantage of AUV mobility to enhance the network lifetime. The trajectory of AUV is elliptical and position of gateways are predefined. The data packets are routed to gateways in multi-hop fashion using the shortest path. The gateways and AUV negotiate and finally the gateways transmit the data packet to the AUV. However, predefined fixed gateways quickly deplete their energy due to the extra burden of relaying data. Moreover, the lack of residual energy threshold mechanism reduces the network lifetime. The improved Adaptive Mobility of Courier Nodes in Threshold-Optimized (iAMCTD) protocol for UWSNs is introduced in [11]. They introduced courier nodes to collect data from nodes and then send the collected data to the surface sink. Courier nodes reduced the communication distance to conserve energy, but at the cost of transmission delay. The authors in [12] investigated efficient data-routing in harsh underwater environments and proposed a routing protocol called Mobicast. They solved the energy hole problem with maximized data collection. In this technique, the network area is divided into 3-D zones, such that AUV gathers data from 3-D zones by moving on a predefined path. The protocol operation is carried out in two phases; data is collected by AUV from a 3-D zone in the first phase and in the second phase, the AUV sends wake-up messages to nodes in the next 3-D zone to avoid topology holes. Nodes in the 3-D zone are allowed to enter in active mode to deliver the data. Therefore, this scheme is successful for overcoming the 3-D coverage hole problem. An energy-efficient data-gathering scheme in underwater wireless sensor networks using a mobile sink is presented in [23]. In this scheme, authors aim to achieve three objectives: improvement of energy conservation, reduction of collision rates using proposed MAC protocol, and proposal of a graph structure for sink mobility.

### 2.4. Delay-Sensitive/Throughput-Based Techniques

3-D geography-based routing protocol is proposed in [24]. The protocol is meant for real- time applications meaning that $D_{E2E}$ is minimized. The 3-D void node problem is also addressed using heuristic techniques. For different types of node density networks, various network parameters are tuned accordingly. In [25], the authors presented an efficient data-delivery scheme for UWSNs. The selection of an optimal path from source to destination plays an important role in efficient data-forwarding. In this technique, a threshold-based fitness function is defined to select the best forwarder. In [26] the authors proposed an energy-efficient depth-based opportunistic routing algorithm with Q-learning (EDORQ) for UWSNs. In their technique, the authors combine a Q-learning technique and opportunistic routing algorithm to improve energy conservation, average network overhead, and packet delivery ratio.

In this paper, we are motivated from the research presented in [12,15]. The Mobicast technique proposed in [12] cannot be adopted for delay-tolerant applications because when the AUV collects data from one 3-D zone the nodes in other 3-D zones remain in sleep mode, causing delay. If there are *n*-zones and AUV spends δ amount of time in each zone, then a total delay of nδ−1 will be created in one round of data collection. Therefore, this technique is inappropriate for delay-intolerant applications. It also affects throughput for intervals of time. The iAMCTD [15] presented a metric for delay-sensitive applications. However, it is not sufficient for energy efficiency and delay minimization for routing in sparse and dense deployment of nodes simultaneously. Selection of forwarder node needs to be modified especially when network becomes sparse due to death of nodes. Table 1 presents similarities and differences between iAMCTD, Mobicast, and DIEER.

## 3. Energy-Consumption Model

In this section, first we define the underwater channel model and then we theoretically analyze energy consumption of the transmitter and receiver nodes.

### 3.1. Channel Model

The SONAR (SOund Navigation And Ranging) equation provides a way of estimating signal-to-noise ratio (SNR) for sound waves underwater. Sound wave propagation underwater is affected by spreading loss, absorption loss, reflection loss, ambient noises, and receiver characteristics. Two types of SONAR equations are used for estimating SNR, Active SONAR and Passive SONAR equations. The former is used for detecting a target, i.e., the intensity of the sound waves returned after hitting the target in the form of echo. The latter is used to detect the intensity of the sound wave received at the destination. We use a passive SONAR equation to detect the SNR ratio for the received sound waves. Sound waves underwater are affected by transmission loss ($T_{L}$). Therefore, the intensity of the received sound waves ($S_{I}$) which is measured in decibels (*dB*) emitted from source level ($S_{L}$), can be calculated from Equation (1)

(1) $$S_{I}=S_{L}-T_{L}$$

where $T_{L}$ is the ratio of signal intensity at source and signal intensity at a distance of one yard from source. It is given by the following equation.

(2) $$T_{L}=10log\frac{I(1yrd)}{I(r)}$$

Similarly, $S_{L}$ is computed as

(3) $$S_{L}=10log\frac{I(1yrd)}{I_{ref}}$$

after substituting the values of $S_{L}$ and $T_{L}$ in Equation (1), we have

(4) $$S_{I}=10log\frac{I(1yrd)}{I_{ref}}-10log\frac{I(1yrd)}{I(r)}$$

By some algebraic manipulations, we have

(5) $$S_{I}=10log\frac{I(1yrd)}{I_{ref}}+10log\frac{I(r)}{I(1yrd)}$$

By further reducing it, we can write as

(6) $$S_{I}=10log\frac{I(r)}{I_{ref}}$$

Apart from these factors, acoustic wave propagation is also affected by ship turbulence, water current movement, wind speed, biological noises, etc. According to the passive SONAR model [27], SNR is given by the following equation.

(7) $$SNR\left(dB\right)=S_{L}-T_{L}-N_{L}+D_{I}\geq D_{T},$$

Values of Noise Level ($N_{L}$), Directive Index ($D_{I}$), and SNR are taken from [28,29]. For deep water, these values are presented in Table 2. $T_{L}$ for deep sea is given by the following equation [30].

(8) $$T_{L}=20\log d+\left(\alpha d\times 10^{-3}\right)$$

Equation (8) shows that $T_{L}$ is directly proportional to distance-dependent attenuation (*d*) and frequency-dependent absorption (α). Thorp’s expression [27] calculates (α) for frequencies above a few hundred Hertz by the following equation.

(9) $$\alpha =\frac{0.1f^{2}}{{\left(1+f\right)}^{2}}+\frac{40f^{2}}{4100+f^{2}}+2.75\times 10^{-4}f^{2}+0.003,$$

For lower frequencies, α is given by Equation (10) as,

(10) $$\alpha =0.11\frac{f^{2}}{{\left(1+f\right)}^{2}}+0.011f^{2}+0.002,$$

where α is measured in *dB/km* and *f* is in kHz.

In our technique, a node can play two roles; Normal node ($N_{n}$) and Forwarder node ($F_{n}$). In the earlier type, it only sends data to $F_{n}$ and in the later type it forwards its own data and collected data to the next $F_{n}$. In the following subsections we analyze the energy consumption of these nodes individually.

### 3.2. Energy Consumption of $F_{n}$

$F_{n}$ collects data in hierarchical way. $F_{n}$ behaves like a parent node and collects data from its child nodes. If $R_{f}$ and $r_{dt}$ are the communication and $d_{th}$ ranges of $F_{n}$, respectively, then distance (*d*) between parent and child node is: $2r_{dt}<d<2R_{f}$. If S is the set of all children nodes (C) of $F_{n}$ then,

$$S=\{C\in S|2r_{dt}<d_{F\to c}<2R_{f}\}$$

If *ℓ*-bits packet is transmitted by each *C*, then the total amount of data gathered at $F_{n}$ is,

(11) $$F_{data}=\sum_{i}^{\left|S\right|}\ell_{i}$$

After collection of data and using Equation (7) we calculate energy consumption of $F_{n}$ as follows,

(12) $$E_{F_{n}}=e_{s}+e_{t}\times R\left(\sum_{i}^{\left|S\right|}\ell_{i}+\ell \right)\phi )+EDA+SNR$$

where $e_{s}$ is the sensing energy, $e_{t}$ is the electronic energy per bit during transmission, ϕ is the data aggregation factor, radius (*R*) and *EDA* is the data-aggregation energy.

### 3.3. Receive Energy of $F_{n}$

**Figure 1:**
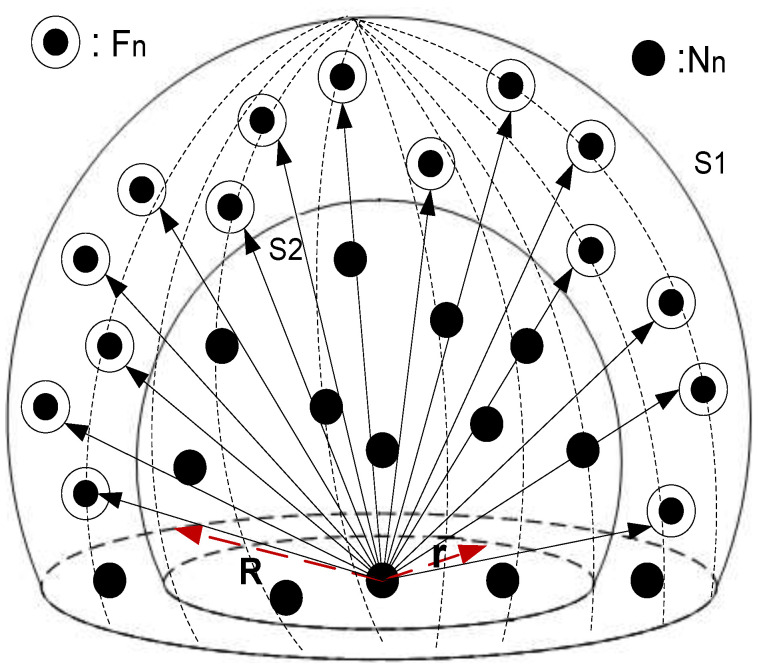
Hemispherical volume of existence of $F_{n}$.

In Figure 1, the communication range of a node is represented in terms of a sphere (S1) with radius (*R*). The depth threshold ($d_{th}$) of a node is represented with another sphere (S2) of radius (*r*). Both S1 and S2 are concentric as shown in Figure 1. In depth-based routing techniques, data travels from high-depth nodes to low-depth nodes; therefore, expected $F_{n}s$ of a $N_{n}$ always exist in the upper hemispherical region of $N_{n}$. Existence of $F_{n}$ is further bounded with $d_{th}$. To find total expected $F_{n}s$ of an $N_{n}$, it is necessary to find the volume of the bounded spherical sector as shown in Figure 1.

Volume of S1 is calculated as follows,

(13) $$\begin{array}{c} V_{S1}=\int_{0}^{r}\int_{0}^{\frac{\pi }{2}}\int_{0}^{2\pi }\varrho^{2}\cos\phi d\theta d\varrho \end{array}$$

(14) $$\begin{array}{c} \begin{array}{c} =\pi \int_{0}^{r}\left(R^{2}-Z^{2}\right)dz\\ =\frac{2}{3}\pi R^{3} \end{array} \end{array}$$

Similarly, the volume of S2 is calculated as follows,

(15) $$V_{S2}=\frac{2}{3}\pi r^{3}$$

By subtracting the volume of S2’s hemisphere from S1’s hemisphere we get the volume of a resultant spherical sector given as,

(16) $$\begin{array}{c} V_{ss}=V_{S2}-V_{S1}\begin{array}{c} =\frac{2}{3}\pi \left(R^{3}-r^{3}\right) \end{array} \end{array}$$

If ψ is the node density of the network then the number of nodes in the bounded spherical segment is given by the following equation.

(17) $$N_{ss}=\frac{2}{3}\pi \psi \left(R^{3}-r^{3}\right)$$

Total received energy consumed by all eligible $F_{n}s$ is,

(18) $$E_{F_{n}-all}^{rcv}=e_{r}+\frac{2}{3}\pi \psi \left(R^{3}-r^{3}\right)\times \ell$$

### 3.4. Energy Consumption of $N_{n}$

Each node when acts as $N_{n}$, it transmits *ℓ*-bits data to $F_{n}$. Therefore, its energy consumption is calculated as:

(19) $$E_{N_{n}}=e_{s}+\left(e_{t}\times R\times \ell \right)+SNR$$

## 4. Calculation of Optimal Radius of Depth Threshold

**Figure 2:**
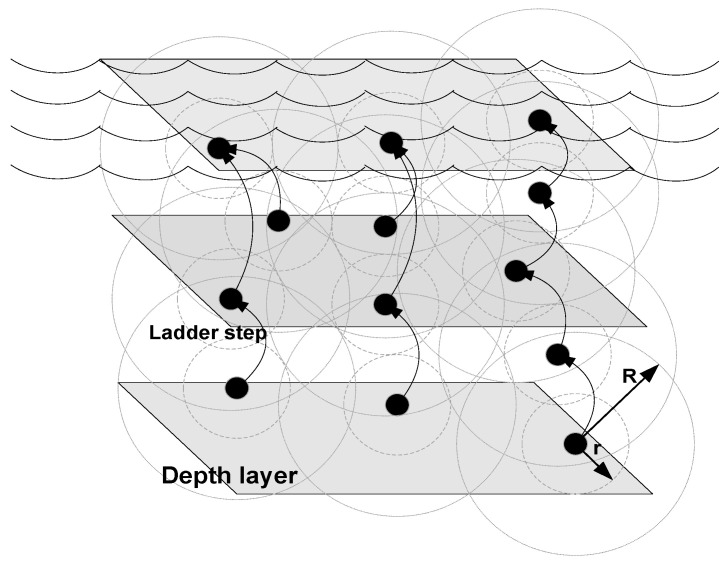
Ladder step analogy of data-forwarding.

In this paper, we assume that nodes are deployed in a 3-D network field as shown in Figure 7. Data travels from nodes on the seabed towards surface sinks through nodes suspended in different layers of sea. Node placement in layers of sea are analogous to ladder steps. Each step of the ladder is considered to be a sea plane as shown in Figure 2. $d_{th}$ is a selection of transmission ranges for each node. There is a tradeoff between $d_{th}$, energy consumption, and propagation delay. Higher $d_{th}$ means higher energy consumption with less propagation delay while lower $d_{th}$ means less energy consumption with large propagation delay. Since each node keeps the list of alive neighbor nodes, in the beginning of the network operation $d_{th}$ remain large. However, as node density reduces due to death of nodes, $d_{th}$ becomes small. Therefore, large $d_{th}$ at the network start-up consumes energy, but at the benefit of smaller propagation delay. On the other hand when sparsity of the nodes increases due to regular deaths then small $d_{th}$ leads to reduced energy consumption. Thus, based on the above discussion, we define $d_{th}$ as

(20) $$d_{th}\left(r\right)=\left\lfloor \left(\Psi -n\right)\%\times R\right\rfloor$$

where *R* is the communication radius of the node, Ψ is the node density of the alive forwarding nodes measured in percent and *n* is the step size, which is considered to be constant. In our case we assume n=20. In the start of operation Ψ=100, this means that $d_{th}\left(r\right)=80\%$ of *R*. However, as the network operation evolves, Ψ decreases say for example 20% nodes die out, and then $d_{th}\left(r\right)=60\%$ of *R* and so on.

## 5. DIEER Protocol Overview

In this research work, we are motivated from [12,15]. The work done in [12] can only be used for delay-tolerant applications because when AUV collects data from one 3-D zone the nodes in other 3-D zones remain in sleep mode. Thus, the AUV spends surplus time in each zone. If there are *n*-zones and AUV spends δ amount of time in each zone then a total delay of nδ−1 will be created in one round of data collection. Therefore, this technique is inappropriate for delay-intolerant applications. It also affects throughput for an interval of time. The metric presented in [15] is not sufficient for energy-efficient routing in the sparse and dense deployment of nodes. The selection of a forwarder node needs to be modified especially when the network becomes sparse due to the death of nodes.

Delay-Intolerant Energy-Efficient Routing (DIEER) is a greedy algorithm with optimized sink mobility that forwards the data packets from source node to destination. The forwarding node’s depth decreases as the packet travels towards the surface sinks. In DIEER, nodes make packet-forwarding decision based on defined priorities, such as the availability of mobile sink in its vicinity, and data-forwarding to minimum depth node. If the depth of the source node is $d_{s}$ and depth of its forwarder node is $d_{f}$ then $d_{s}<d_{f}$ otherwise forwarder node simply drops the packet.

### 5.1. Adaptive Depth Threshold Mechanism (ADTM)

**Figure 3:**
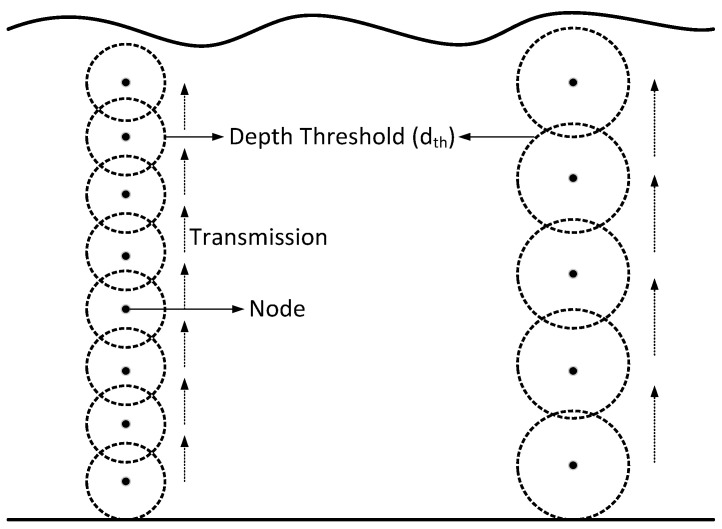
Varying depth threshold in DBR.

The data packet broadcast by the source node is received by multiple nodes within *R* of source node; therefore, if all eligible nodes forward the same packet, high energy consumption and high collision will occur. To reduce the transmission of same packets by multiple nodes, the number of forwarder nodes needs to be restricted. According to ADTM, each node selects a set of neighboring nodes within *R* and *r*. Variation in *r* of $d_{th}$ depends upon the number of alive nodes in the vicinity of the source node. If a greater number of alive nodes exist within *R* of the source node, then *r* of $d_{th}$ increases and vice versa. Figure 3 depicts the comparison of small $d_{th}$ with optimal $d_{th}$. It is shown in Figure 3 that when $d_{th}$ is small the number of transmission(s) are increased which leads to increased energy consumption because when multiple nodes receive and transmit the same data packet the overall energy consumption increases. However, for optimal $d_{th}$ the transmission(s) and reception(s) decrease which ultimately reduces the energy consumption.

### 5.2. Hold and Forward Mechanism (HFM)

**Figure 4:**
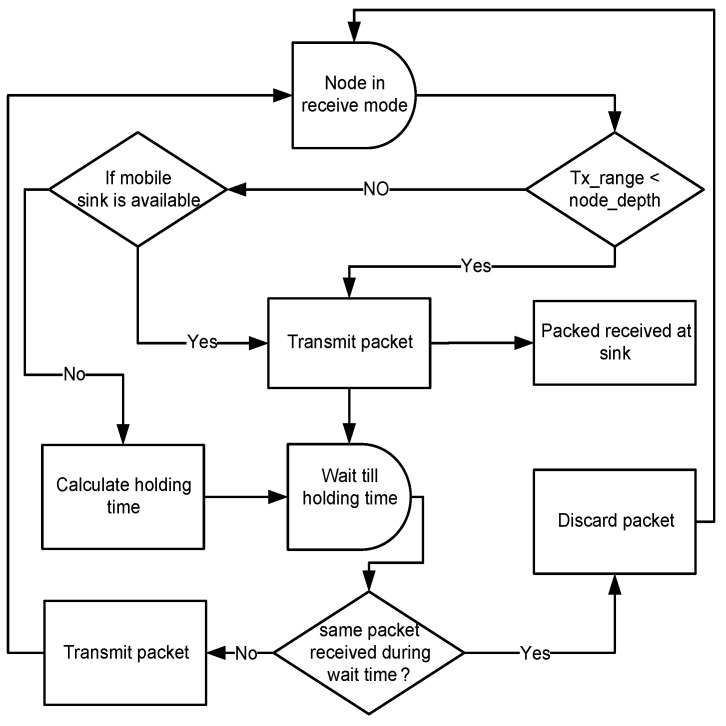
Hold and forward mechanism of packet-forwarding.

**Figure 5:**
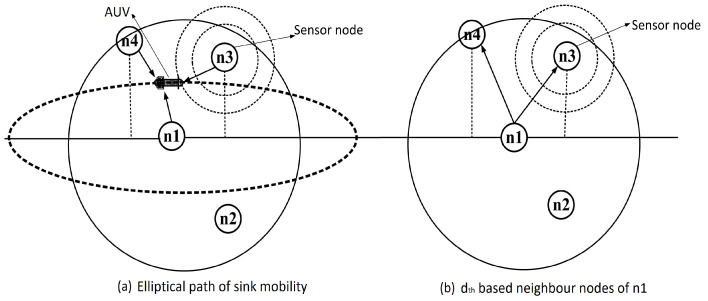
ADTM with sink mobility.

In DIEER, the node forwards the data packet by using a Hold and Forward Mechanism (HFM) mechanism. HFM is depicted in Figure 4. Each receiving node holds the data packet for a certain interval of time known as Holding Time (HT).The calculation of HT depends on the depth difference between the receiving node and the transmitting node. If the depth difference is large then the packet holding time is minimum and vice versa. HT and Depth Difference (DD) are inversely proportional to each other (HT∝1/DD). During HT, if a node finds a mobile sink in its vicinity then the first data packet is transmitted to the mobile sink. Figure 5a shows an elliptical path of sink mobility in which the mobile sink is in direct communication range of node $n_{1}$, $n_{2}$ and $n_{3}$. In this case, the nodes first calculate their distance from the mobile sink, if their distance with the mobile sink is less as compared to next forwarder node then it simply transmits the data packet to the mobile sink.

If the mobile sink is not available, then the data packet is forwarded to the next hop node. For example, $n_{1}$ in Figure 5b is the transmitter node and $n_{2}$, $n_{3}$, $n_{4}$ are its one-hop threshold-based neighbor. The solid line shows the transmission range of $n_{1}$. When $n_{1}$ broadcasts a packet, all its neighbor nodes receive this packet. As $n_{2}$ is at lower depth than $n_{1}$, it discards the packet. $n_{3}$ and $n_{4}$ are eligible forwarders because these are at higher depth than $n_{1}$. The depth of $n_{4}$ is less than $n_{3}$, thus, HT of $n_{4}$ is less than $n_{3}$.

### 5.3. Data Aggregation with Pattern Matching

A sensor node by its nature sense the environmental parameters periodically as per requirement. In data-sensitive applications, the sensing time span is very short. Therefore, nodes record plenty of redundant data of similar values. To reduce the redundancy, data aggregation is required. Several data-aggregation techniques are introduced in the literature [31]. Pattern matching is a popular data-aggregation technique [32]. To improve energy conservation, we use Algorithm 1 for pattern matching. Before transmitting the data packet, each node executes Algorithm 1 to generate a pattern code. Pattern codes are generated up to a predefined threshold value. If the generated code is equal to or greater than the threshold value, then data is sent as it is; otherwise, only a pattern code is embedded in the packet. The pattern-matching algorithm helps to reduce energy consumption remarkably, especially at forwarder nodes, where more than one node’s data is aggregated and forwarded.
**Algorithm 1** Data Aggregation with Pattern Matching. INITIALIZATIONS α= thresholdvalue code =α p_code = Generate_pattern_code(data,code) END INITIALIZATIONS int Generate_pattern_code(data,code) { data_matched = Compare_data_with_stored_pattern(data,code) **if** (data_matched) **then**   return (code) **else**   return (α) **end if** } END Generate_pattern_code **if** (p_code ==α) **then**   send_data(data) **else**   send_data(p_code) **end if**


## 6. Network Lifetime Maximization and $D_{E2E}$ Minimization Models

Since energy-efficient and delay-intolerant protocol designs are our main concerns, and there are many ways/techniques of doing so. In this regard, we have exploited the network layer. However, to further improve energy efficiency of the network and to down-scale $D_{E2E}$, we present formulation via linear programming. The first objective, to maximize the network lifetime NL, is as follows:

(21) MaxNL

Subject to,

(22a) $$\begin{array}{c} E\left(i\right)\leq E_{0}\;\;\; \end{array}$$

(22b) $$\begin{array}{c} \sum_{i}l\left(i\right)\left(E_{sen}\left(i\right)+E_{rec}\left(i\right)+E_{tra}\left(i\right)+E_{da}\left(i\right)\right)\leq qE\left(i\right) \end{array}$$

(22c) $$\begin{array}{c} f(i,j)\leq C(i,j) \end{array}$$

(22d) $$\begin{array}{c} n_{re-tx}⟶0 \end{array}$$

(22e) $$\begin{array}{c} Min\;\;\;n_{hops} \end{array}$$

where $NL=\sum_{r}t\left(r\right)$ and $t\left(r\right)=\frac{\sum_{i}E\left(i\right)}{\sum_{i}l\left(i\right)\left(E_{sen}\left(i\right)+E_{rec}\left(i\right)+E_{tra}\left(i\right)+E_{da}\left(i\right)\right)}$. Here, t(r) provides a formal explanation about the involved processes; sensing ($E_{sen}$), transmission ($E_{tra}$), reception ($E_{rec}$), and data aggregation ($E_{da}$). The objective function is presented in Equation (21), i.e., to maximize the NL. Constraint in Equation (21a) states that each node is initially equipped with limited energy E(i) upper bounded by $E_{0}$. Constraint in Equation (21b) jointly considers sensing, reception, transmission, and aggregation while ensuring that these processes respect their initial levels, such that $q=\frac{1}{NL}$. Our proposed DIEER protocol uses optimal $d_{th}$ and mobile sink as tools to minimize the number of transmissions, receptions, and aggregator nodes to minimize energy-consumption cost due to these processes. Constraint in Equation (21c) ensures flow conservation (i.e., flow through the link from node *i* to node *j* must respect the physical link capacity C(i,j)) while routing data from source node to sink. Violation of (21c) leads to increased contention/congestion which is directly related to both packet drop rate and $D_{E2E}$. Whenever the dropped packets are re-transmitted, the surplus energy is spent leading to decreased NL. Constraint in Equation (21d) focuses on the minimization of re-transmissions to further down-scale the energy-consumption cost. The proposed DIEER protocol implements holding time to respect constraints in Equations (21c,d). Finally, constraint in Equation (21e) deals with minimization of the number of hops $n_{hops}$ to maximize NL. The proposed DIEER protocol efficiently uses mobile sink and optimal $d_{th}$ to minimize $n_{hops}$.

**Graphical analysis:** Consider a scenario in which a source node *s* intends to communicate with sink via intermediate node *i* such that the transmit power is 0.524 W and aggregation factor at node *i* is 0.6. Energy-consumption cost in transmission and reception is $2\times P_{tx}$ and $0.1\times P_{tx}$, respectively. The transmit and receive power costs are for a transmission range of 100 m. If we vary the transmission range between 10–100 m, the energy-consumption costs (with units in joule) of source node and intermediate node can be modeled via the following set of equations.

(23) 0.01048≤E(s)≤1.048

(24) 0.01132≤E(i)≤1.132

(25) 0.0218≤E(s)+E(i)≤2.18

**Figure 6:**
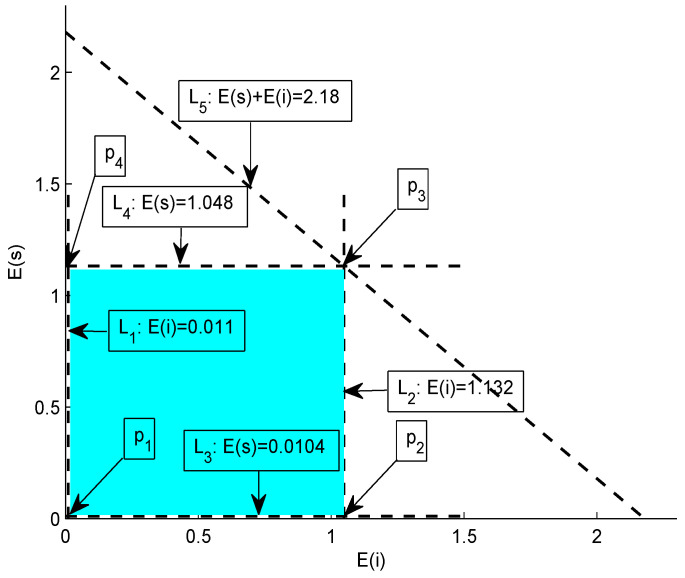
Feasible region.

Subject to the upper and lower bounds provided by Equations (24)–(26), Figure 6 shows the intersection of five lines; $L_{1}$ to $L_{5}$. These lines result in the formation of a bounded region called feasible region. This region contains the set of all possible solutions, i.e., each point that within this region represents a valid solution. Now, we test each vertex of the feasible region,

at $p_{1}$: 0.01132+0.01048=0.0128J,
at $p_{2}$: 1.132+0.01048=1.1424J,
at $p_{3}$: 1.132+1.048=2.18J,
and at $p_{4}$: 0.01132+1.048=1.0593J.

Hence, it is proved that all the valid energy-consumption costs of source and intermediate nodes for the given specifications lie within the illustrated feasible region which is colored cyan in Figure 6.

As mentioned earlier, we aim to delay-intolerant applications. Thus, in our second objective, $D_{E2E}$ minimization is formulated as follows,

(26) $$Min\;\;\;D_{E2E}\left(S,MS\right)$$

where

(27) $$\begin{array}{c} D_{E2E}\left(S,MS\right)=\left\{\begin{array}{cc} D\left(S\right)+D_{E2E}\left(j,MS\right) & \mathrm{if}\;j=MS\\ D(S) & \mathrm{if}\;j=MS \end{array}\right. \end{array}$$

and

(28) $$D\left(S\right)=D_{tra}\left(S\right)+D_{queue}\left(S\right)+D_{re-tra}\left(S\right)$$

(29) $$D\left(j\right)=D_{rec}\left(j\right)+D_{agg}\left(j\right)+D_{tra}\left(j\right)+D_{re-tra}\left(j\right)$$

(30) $$D\left(MS\right)=D_{rec}\left(MS\right)+D_{agg}\left(MS\right).$$

Subject to,

(31a) $$\begin{array}{c} 0\leq |N|\leq a\;\;\; \end{array}$$

(31b) $$\begin{array}{c} p\left(i\right)\leq C_{pkt}\left(j\right) \end{array}$$

(31c) $$\begin{array}{c} \lambda (i)\leq \mu (i) \end{array}$$

Equation (26) presents the objective function, i.e., to minimize $D_{E2E}$ from source node *S* to the mobile station MS. Equation (27) defines the $D_{E2E}$ for the two possible cases; node(s) are involved in data routing and the intermediate node(s) are not involved in the data-forwarding. In the former case, the total $D_{E2E}$ when *S* communicated with MS includes nodal delay at *S* (i.e., D(S)) and $D_{E2E}$ from intermediate node *j* to destination MS (i.e., $D_{E2E}\left(j,MS\right)$). Equations (28)–(30) provides details about the delay contributors at *S*, *j* and MS, respectively. Where $D_{tra}$ denotes transmission delay, $D_{queue}$ represents queuing delay, $D_{re-tra}$ is the re-transmission delay, $D_{rec}$ denotes reception delay and $D_{agg}$ is the data-aggregation delay. Constraint in Equation (31a) bounds the number of nodes ∣N∣ between 0 and *a*. If the network is dense, then relatively high number of nodes contend for channel access that leads to increased $D_{E2E}\left(S,MS\right)$ (referred Equations (27)–(30)). Constraint in Equation (31b) states that the packet sent from node *i* (i.e., p(i)) must not exceed the packet-handling capacity $C_{pkt}$ at node *j*. Violation of (31b) leads to congestion at node *j* causing unbounded $D_{queue}$. Finally, constraint in Equation (31c) means that the arrival rate λ at a given node must not exceed the departure rate μ at that particular node, otherwise, $D_{queue}$ will increase if (31b) is not violated, else $D_{re-tra}$ will rise.

## 7. Simulation Results and Analysis

This section discusses the performance analysis of our proposed protocol in comparison with Mobicast and iAMCTD. Simulation parameters are given in Table 3. Existing MAC solutions for terrestrial WSN are not suitable for UWSN because of low propagation, high bit error rate, low bandwidth, multi-path and fading phenomena. Currently MAC solutions for UWSN are based on CSMA or CDMA protocols. However, for real implementations we assume to use UW-MAC introduced in [33], because UW-MAC guarantees low energy consumption, high throughput and low channel access delay. UW-MAC influences CDMA properties for medium access. UM-MAC does not use a hand-shaking mechanism (RTS/CTS) therefore, reduces collision and increases channel reusability. Moreover, to further reduce the delay produced due to the back-off contention mechanism the size of the contention window ($CW_{min}$ and $CW_{max}$) is reduced to 8 to 64 which was originally set as 32 to 1024. For simulation purposes, we assume a collision-free underwater wireless channel, therefore, interference effects in the wireless channel are ignored.

**Figure 7:**
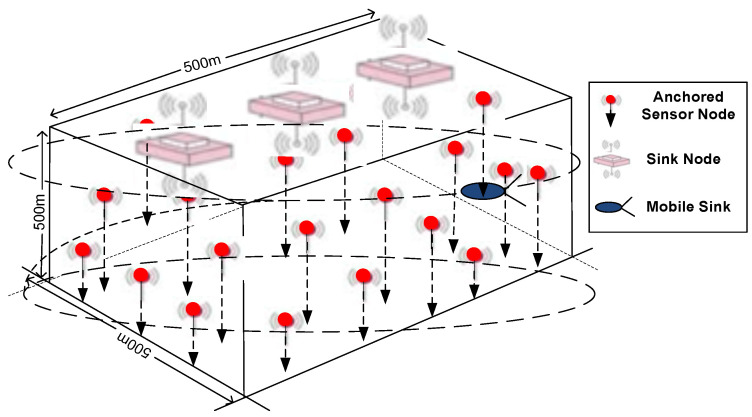
Deployment model.

In UWSN, sensor nodes collaboratively perform an ocean column monitoring task over a 3-D target area. Therefore, in this research work, we consider the nodes are randomly deployed and are anchored with wires and floating mechanism [34]. Sinks float on the surface of the sea having dual capability of communication with the offshore control room, mobile sink and sensor nodes. The mobile sink moves around the three-dimensional network field in elliptical spiral paths to collect data from sensor nodes and transmit it to surface sinks. An overview of the deployment strategy is shown in Figure 7.

For simulation purposes, we used characteristics of an acoustic modem described in [35]. According to the characteristics of the modem, it consumes 2.5 mW(milli Watt) power in stand-by mode, 5–285 mW power in listening mode, and 1.1 mW power in receive mode. For transmission mode the modem has three ranges: for 250 m range it consumes 5.5 W, for a range of 500 m modem consumes 8 W, and for 1000 m it consumes 18 W. In our simulations, we have downscaled these values for 10 m, 50 m, and 100 m ranges. Table 3 shows the environment parameters defined for simulation and Table 4 shows the performance metrics used for assessment of the simulation results. To simulate redundant (duplicate) data generation, we generated random data of variable lengths with 10%, 20% and 30% duplication.

### 7.1. $D_{E2E}$

**Figure 8:**
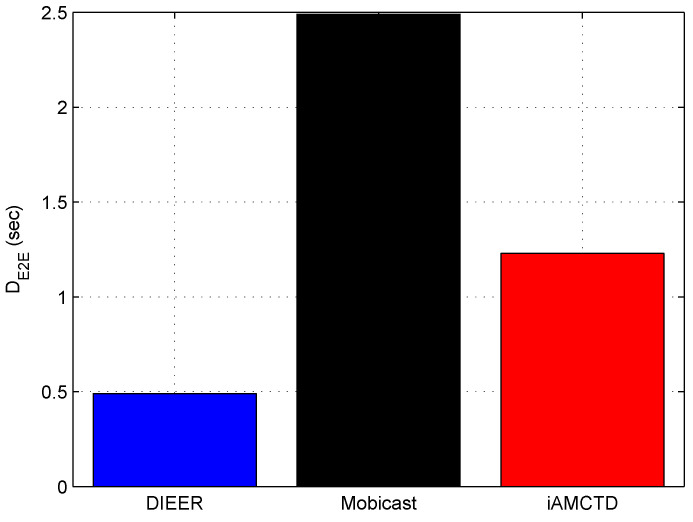
Average $D_{E2E}$.

Figure 8 shows that DIEER has significantly reduced $D_{E2E}$ as compared to Mobicast and iAMCTD. Among the three compared protocols, Mobicast attains the highest $D_{E2E}$. DIEER’s $D_{E2E}$ is *80%* reduced than Mobicast. In Mobicast, mobile sink moves on a specified path to collect data. Therefore, nodes remain in sleep mode and do not transmit data until mobile sink reaches them to collect data. Thus, a sleep–awake mechanism creates surplus $D_{E2E}$ in the network. Moreover, the relatively larger travel distance and greater number of hops in Mobicast contribute in delay as well. On the other hand, iAMCTD has relatively reduced $D_{E2E}$ due to courier nodes. However, the number of hops in iAMCTD are greater in number than DIEER, thereby, iAMCTD has a higher $D_{E2E}$ than DIEER. To sum up, DIEER achieves the least $D_{E2E}$ among the three compared protocols shown in Figure 8 due to three reasons: (i) reduced communication distance; (ii) efficient usage of mobile sink; and (iii) reduced number of transmissions and receptions.

### 7.2. Throughput

**Figure 9:**
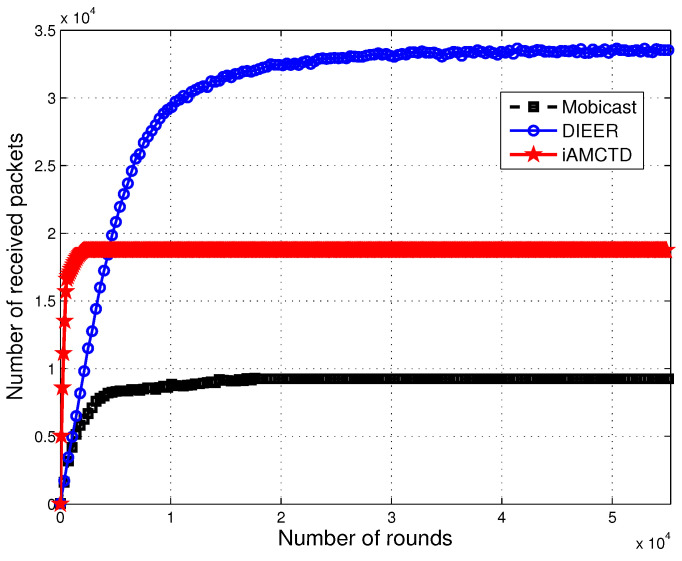
Network throughput.

Different techniques are proposed at physical and routing layer to maximize throughput. However, it is not always possible to successfully deliver every data packet at destination due to drastic and harsh environments of underwater. We have used the uniform random model [36] to calculate the number of dropped packets. In DIEER, the availability of sinks has a higher probability than Mobicast. In Mobicast, mobile sink (AUV) is the only source of data forwarder to surface sink. In DIEER, mobile sink and multiple forwarder nodes (acting as sink) are the multiple sources of data-forwarding to surface sink. As there are multiple forwarder nodes, therefore, the availability of the sink has higher probability in DIEER as compared to Mobicast. DIEER adopts this design of data-forwarding to reduce the wait time of the mobile sink. However, Mobicast lags behind DIEER in this case because the mobile sink in Mobicast moves in the entire network field and collects data. So, the mobile sink at time (t) will be available in a specific zone only, while nodes of other zones are in wait state. Moreover, mobile sink of DIEER decreases the communication distance, which leads to more reliable communication. On the other hand, courier nodes of iAMCTD are advantageous in this regard as well. Thus, throughput of iAMCTD is greater than Mobicast. However, both iAMCTD and Mobicast have smaller throughput than DIEER as shown in Figure 9.

### 7.3. Energy Consumption

**Figure 10:**
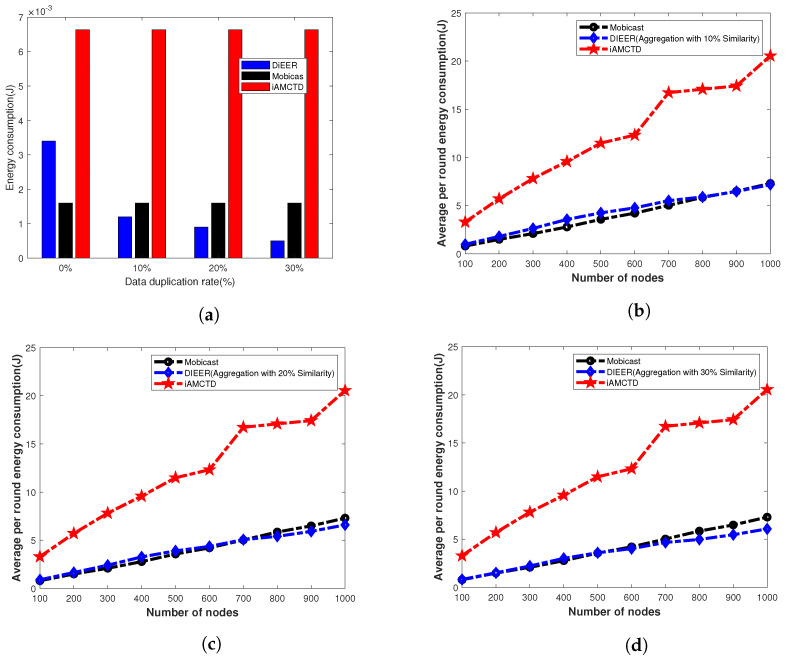
Energy-consumption analysis. (**a**) Energy-consumption analysis with varying data duplication rate; (**b**) Energy consumption with 10% data duplication; (**c**) Energy consumption with 20% data duplication; (**d**) Energy consumption with 30% data duplication.

Energy consumption mainly depends upon packet size and the transmission distance between source and destination. Our protocol uses Algorithm 1 to avoid duplicate data transmission. To evaluate energy consumption of the three protocols, we run the simulations in two different scenarios. In first case, we run the simulation for fixed number of nodes and data duplication rate vary with 0%, 10%, 20% and 30% as shown in Figure 10a. In second case, data duplication as well as number of nodes vary from 100 to 1000 as shown in Figure 10b–d. From Figure 10a, it is clear that energy consumption of Mobicast with 0% duplication is better than other two protocols because most of the time nodes remain in sleep mode. Nodes wake up for transmission only when AUV arrives in their transmission range. Therefore, Mobicast consumes less energy. However, as we introduce duplicate data in the simulations, the energy consumption of DIEER starts improving due to Algorithm 1, because DIEER starts suppressing duplicate data which conserves energy. Figure 10b–d shows that energy consumption of DIEER significantly improves as the number of nodes increase.

Comparative analysis of these figures show that energy consumption of iAMCTD increases as number of nodes increase. This is because iAMCTD uses fixed $d_{th}$ for transmission and re-transmissions. Energy consumption of DIEER reduces with increase in number of nodes. Low energy cost of DIEER is because of its adoptive $d_{th}$ mechanism and suppression of duplicate data transmission. As the number of nodes increases $d_{th}$ increases, which ultimately restricts nodes to involve in re-transmission and Algorithm 1 helps to reduce packet size by just sending data pattern code for duplicate data.

### 7.4. Network Lifetime

**Figure 11:**
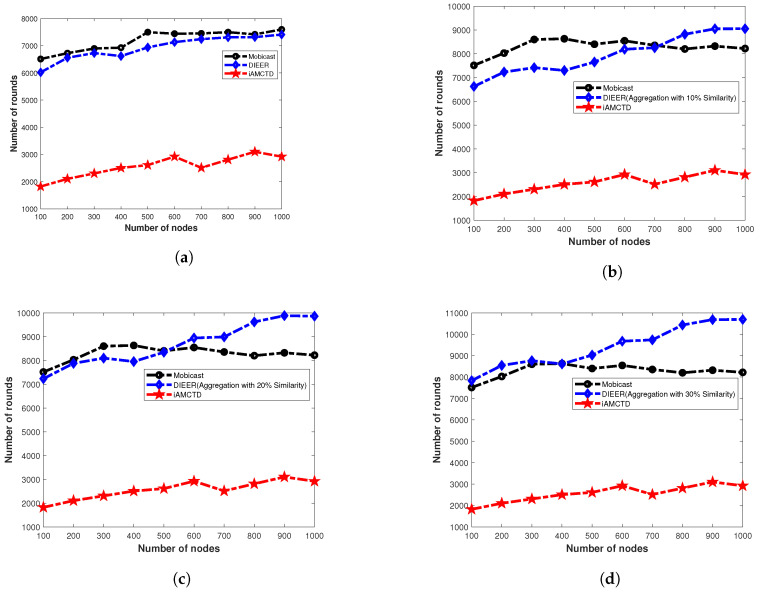
Network lifetime analysis. (**a**) Network lifetime without Aggregation; (**b**) Network lifetime with 10% data duplication; (**c**) Network lifetime with 20% data duplication; (**d**) Network lifetime with 30% data duplication.

Figure 11a shows the NL of three compared protocols; Mobicast, iAMCTD and DIEER with varying number of nodes and without data aggregation. In this Figure 11a, NL of Mobicast is better than DIEER and iAMCTD. However, in Figure 11b–c as the data aggregation with pattern matching is introduced the NL of DIEER improves significantly over Mobicast and iAMCTD. This is because in DIEER, nodes forward data packet by using HFM mechanism. Each receiving node holds the data packet for a certain interval of time known as HT. Calculation of HT depends on depth difference between receiving node and the transmitting node. If DD is large then HT is minimum and vice versa. HT and DD are inversely proportional to each other (HT∝1/DD). In DIEER, $d_{th}$ is used for the selection of forwarder nodes for re-transmission, i.e., increase/decrease of communication radius of a node. Larger radius ($d_{th}=80$) means selection of farthest node as forwarder node which reduces propagation delay (due to involvement of minimum hops) and restricts maximum nodes to involve in re-transmission additionally pattern-matching data-aggregation mechanism of DIEER reduces packet size, thus both mechanisms save overall network energy and prolong network lifetime.

### 7.5. Scalability Analysis

**Figure 12:**
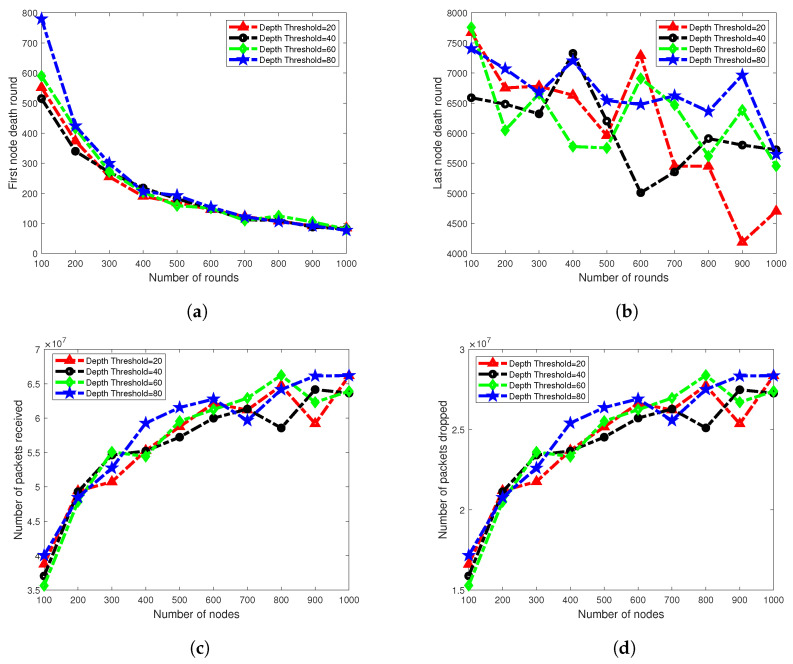
Scalability Analysis. (**a**) First node died analysis; (**b**) All nodes died analysis; (**c**) Packets received analysis; (**d**) Packets dropped analysis.

Network scalability is an important factor for measuring the performance of the routing protocol. With the help of this metric, we can estimate the performance of a routing protocol from different perspectives for example, if number of sensor nodes in the network grows larger than how the protocol behaves in terms of throughput, delay, lifetime, etc. To measure the scalability performance of our protocol, we deploy *100* to *1000* nodes with an incremental step of *100* nodes, randomly in the network field. As in our scheme, selection of threshold radius is adaptive, therefore, to see the effect of varying threshold radius we performed simulations for *20* m, *40* m, *60* m and *80* m of radii. Rest of the simulation settings remain same as mentioned in Table 3. In Figure 12b, last node death round is shown against various number of nodes and $d_{th}$ radius. Result shows that network lifetime remains stable for $d_{th}=80$. There is a little variation for *100* to *1000* nodes. However, network lifetime of $d_{th}=20$ is seriously affected. It fluctuates from *6400* rounds to *4000* rounds for *100* to *1000* nodes, respectively. The main reason for reduced network lifetime is high energy consumption due to the received energy-consumption process.

Figure 12a shows the first node died time for various numbers of nodes. Result shows that the network performance decreases as the number of nodes is increased. In our scheme, the radius of the $d_{th}$ mechanism identifies the involvement of a number of nodes in packet-forwarding. A smaller number of nodes and larger radius of threshold means a fewer number of nodes are involved in packet-forwarding, which creates fewer interference effects among nodes and less energy will be consumed in the receiving process. However, on the other hand, many nodes with small radius of $d_{th}$ creates high energy consumption in the network, which results in rapid network instability as shown in Figure 12b. To overcome this problem, our protocol uses ADTM.

Behavior of the graph in plots of packet received and packets dropped, shown in Figure 12c,d, respectively, is stable. Plots of all four $d_{th}$ types are showing an increasing trend, because a larger number of nodes are involved in the network operation; therefore, the packets received ratio is also increased. Similarly, as a greater number of packets are sent to BS, the chance of packet drops is also increased. From the scalability analysis of $d_{th}$ and number of nodes, we conclude that our protocol is scalable to some extent for $d_{th}=80$.

## 8. Conclusions

In this research work, we presented an energy-consumption model for depth-based routing protocols. Then, we introduced a metric for calculation of optimized $d_{th}$ with varying node density in the network. In our technique, data packets are continuously transmitted, therefore the concept of pattern-matching-based data aggregation is introduced to conserve energy. Additionally, a hybrid approach of mobile sink and surface sink is introduced. A mobile sink moves in a 3-D elliptical path to enhance the network throughput and minimize $D_{E2E}$. In this research work, we have addressed the low data-delivery ratio and high $D_{E2E}$ problems of Mobicast. Simulation results prove that our protocol performed well in drastic condition(s) in terms of data-gathering as compared to Mobicast. Moreover, scalability analysis shows that our protocol is scalable for $d_{th}=80$. 

## Figures and Tables

**Table 1:** Similarities and differences.

	iAMCTD	Mobicast	DIEER
**Deployment** **Strategy**	Random, 3D deployment for monitoring ocean column sensing	Random, 3D deployment for monitoring ocean column sensing	Random, 3D deployment for monitoring ocean column sensing
**Data collection**	Through mobile Sink DBR-based, through forwarder nodes	Through mobile Sink	Through mobile Sink DBR-based, through forwarder nodes
**Data-forwarding** **mechanism**	DBR-based Holding time calculation based	Sleep–awake mechanism through mobile sink	DBR-based Holding time calculation based
**Holding time calculation**	Based on node’s depth Forwarding function metric-based	No holding time calculation	Based on node’s depth Depth threshold-based
**Sensing mechanism**	Periodic regularsensing	Periodic regularsensing	Periodic regularsensing
**Sink mobility pattern**	Initially wide elliptical paths In sparse conditions narrow elliptical paths	Apple slice technique used to cover all 3D zone of reference	Regular fixed elliptical paths
**Data Aggregation**	Simple	Simple	With pattern matching
**Addresses time** **critical applications**	Yes	No	Yes
**Performance Evaluation** **Parameters**	Average energy consumption End-to-end delay Throughput Transmission loss Network life time in terms of alive nodes and dead nodes	Average energy consumption End-to-end delay Through put Successful packet delivery ratio	Average energy consumption End-to-end delay Throughput Network life time in termsof alive nodes and dead nodes Scalability Analysis for: ⚬ Network lifetime ⚬ Throughput ⚬ Energy consumption

**Table 2:** Physical layer model.

Parameter	Value
$N_{l}$	50 dB
$D_{I}$	3 dB
SNR	20 dB

**Table 3:** Simulation parameters.

Parameter	Value
Network size	500 m × 500 m × 500 m
Number of nodes	250
Initial energy of normal nodes	60 J
Data-aggregation factor	0.6
Packet size	125 bytes
Transmission range of node	100 m
Number of mobile sinks	1

**Table 4:** Network performance parameters.

Performance Parameters	Definition
$D_{E2E}$	It is defined as the time taken by data packet to reach from source to sink. It is measured in seconds.
Throughput	It is defined as total number of data packets successfully received at sink. It is measured in packets per unit of time.
Energy Consumption	It is average amount of energy consumed during one round. It is measured in joules.
NL	It is the time duration from the start of network till the death of last node. It is measured in rounds.
